# Design and validation of an automated low-cost system for real-time gas quantification in bioreactors using pressure sensors

**DOI:** 10.1007/s00449-026-03334-6

**Published:** 2026-04-18

**Authors:** Rodrigo Santos Souza, Alessandra Giordani, Rafael Brito de Moura, Laos Alexandre Hirano, Rafael de Oliveira Tiezzi

**Affiliations:** 1 Institute of Science and Technology—Unifal-MG—Federal University of Alfenas, Cidade Universitária, José Aurélio Vilela Road, 11999, (BR 267 Km 533), Poços de Caldas, MG CEP 37715-400 Brazil; 2 Nature Center Science, University Federal of São Carlos, Campus Lagoa do Sino, Lauri Simões de Barros Road, km 12 - SP-189, Aracaçu, Buri, SP Brazil

**Keywords:** Low-cost pressure sensors, System design and calibration, Gas measurement, Anaerobic biodigestion, Gompertz model

## Abstract

Accurate and reliable gas quantification is essential for evaluating biochemical processes in laboratory-scale reactors, yet traditional measurement methods often lack automation, require extensive manual operation, and involve high implementation costs. This study presents the design, calibration and validation of a low-cost, fully automated system for real-time gas quantification in benchtop bioreactors. The novelty of this work lies in developing a pressure-based measurement approach that ensures accuracy, repeatability and reproducibility while enabling continuous and traceable monitoring of gas production under controlled laboratory conditions. The system integrates MPX5700DP pressure sensors with an Arduino microcontroller, allowing uninterrupted pressure measurement and data logging. Calibration was performed by injecting known air volumes, yielding linear responses with R² > 0.99. System performance was validated through fermentation assays using glucose and yeast, and the resulting pressure data were converted to gas volume and fitted to a modified Gompertz model to extract kinetic parameters, including maximum gas production, production rate, and lag phase. The influence of pH and temperature on system performance was assessed through a Central Composite Design, confirming its robustness across different operational conditions. This setup offers a cost-effective, accurate, and replicable alternative to conventional gas quantification methods, improving automation, precision, and accessibility in research laboratories.

## Introduction

Real-time pressure measurement is a critical tool for accurately monitoring gas production in laboratory-scale experiments. To evaluate the performance of such measurement systems, anaerobic biodigestion was selected as a relevant application due to its importance in treating organic-rich waste streams, such as municipal solid waste, agricultural residues, industrial effluents, and sewage sludge, while producing biogas comprising methane, carbon dioxide, and hydrogen sulfide [1, 2]. Most experiments investigating anaerobic biodigestion are conducted at the laboratory scale, where conditions such as pH, substrate composition, and temperature can be carefully controlled. Anaerobic biodigesters function as biological reactors in which microorganisms degrade organic substrates, reducing organic matter and generating measurable gas volumes. This controlled laboratory environment provides an ideal setting for testing the accuracy, repeatability, and reproducibility of pressure sensors, ensuring reliable and traceable measurements under realistic conditions [2, 3].

A key method for assessing the performance and potential of anaerobic biodigestion systems is laboratory testing, commonly referred to as Biochemical Methane Potential (BMP) assays. The initial procedures for BMP tests involve assessing the anaerobic biodegradability of a sample by comparing gas production in flasks containing the substrate with control flasks and the lack anaerobic activity [4]. While these tests are critical for evaluating biogas potential, traditional BMP methods and automated systems often involve high equipment costs, which limits their accessibility. To address this limitation, recent research in the field has focused on developing cost-effective alternatives that enable broader implementation, lower experimental costs, and reduce measurement errors.

Typical volume displacement devices, such as the Mariotte bottles, eudiometer tubes, and lubricated syringes, are inexpensive and widely used. However, these intermittent meters are labor-intensive, requiring periodic supervision and resetting, which limits their use in long-term experiments. They are better suited for measuring accumulated volumes and may not capture transient variations in gas production rates. Closed-bottle manometric techniques, which rely on pressure increases to estimate biogas volumes, are more sensitive to low production levels but can underestimate gas volumes due to altered gas solubility at elevated pressures. These methods are also rarely suitable for continuous-flow reactors. Semi-continuous gas flow meters that periodically release known gas volumes, such as those using electrically actuated valves or inverted tipping buckets, offer a practical alternative. Thus, selecting an appropriate gas measurement system requires considering factors such as accuracy, reliability, cost, ease of maintenance, and applicability across different flow rates [5].

Several methods for biogas measurement have been developed, such as the approach by Aquino et al. [6], which used alkaline solution displacement for indirect methane measurement. However, the use of graduated cylinders introduces significant errors, leading to cumulative inaccuracies. More advanced and low-cost systems have also been explored, such as Arduino-based solutions with TGS2600 sensors [7] and transducer-based systems for pressure measurement [8, 9]. For instance, Araujo and Oliveira (2014) [5] designed an open-source, lab-scale biogas flow meter featuring data-logging capabilities. Their approach utilized low-cost and readily available electronic components. The flow meter was calibrated, tested, and validated during batch biogas production experiments, where it was compared to a commonly used commercial device. The semi-continuous meter achieved a resolution of 7.45 ± 0.13 mL per pulse and maintained a consistent pulse volume (with less than 2% relative standard deviation) across flow rates from 60 mL.h⁻¹ to 1120 mL.h⁻¹. This robust, low-cost, and dependable system can be easily replicated, modified, and upgraded in laboratories worldwide.

Accurate and reliable gas measurement is essential for monitoring biochemical processes and validating laboratory experiments. Traditional methods, such as volume displacement devices and semi-continuous flow meters, are often indirect, labor-intensive, and less precise, limiting their accessibility, particularly for research groups with restricted funding. These methods also face challenges in capturing transient variations and providing continuous data, which are critical for detailed process analysis. While low-cost alternatives exist, further advancements are needed to improve accuracy, reproducibility, repeatability, and traceability in laboratory-scale gas measurement systems.

In this context, the present study proposes a low-cost, fully automated pressure-based system capable of converting pressure changes into volumetric gas measurements in real time. The system integrates commercially available pressure sensors with a microcontroller platform, enabling continuous monitoring, high sensitivity, and reliable data acquisition. The device was calibrated and validated under controlled laboratory conditions, with explicit evaluation of measurement uncertainty, repeatability, reproducibility, and error propagation. Its performance was further assessed using Biochemical Methane Potential (BMP) assays, analyzing methane production kinetics through the Gompertz model [10] within a Central Composite Design (CCD) framework [11].

By providing an accessible, robust, and metrologically rigorous tool for gas measurement, this work advances the field of measurement science, offering a practical solution that combines low cost, automation, and high reliability, while also supporting laboratories with limited resources.

## Materials and methods

### Real-time pressure development monitoring and integration of the module into bioreactors

**Fig. 1:**
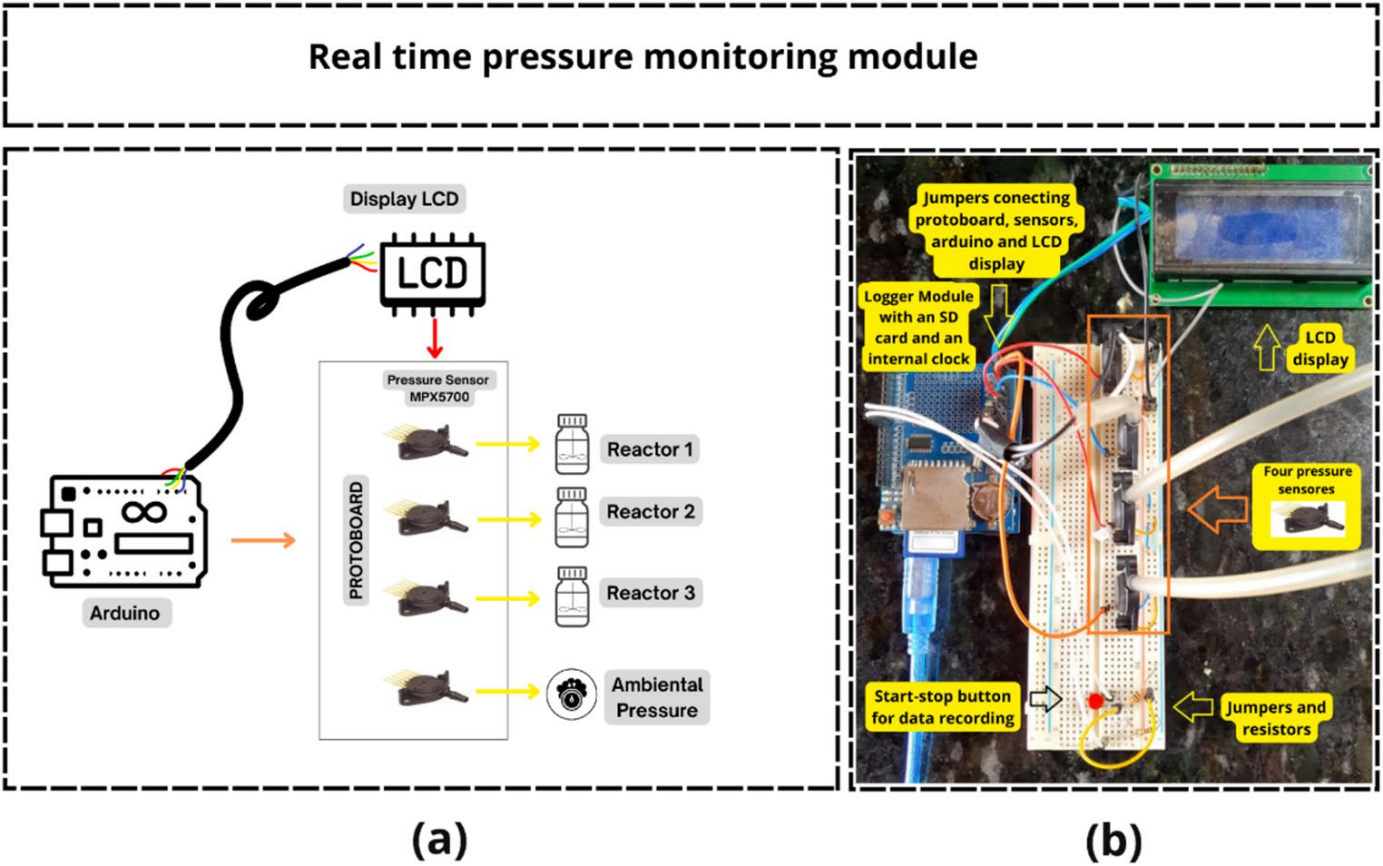
(**a**) schematic model of the experimental apparatus; (**b**) picture of the developed instrumentation

A system for measuring gas production was designed by integrating pressure sensors with microcontrollers for continuous and real-time monitoring. The system utilizes the MPX5700DP pressure sensor module (Fig. 1) to detect pressure differences within the experimental setup, adapting the manometric method described by McEniry and O’Kiely [9].

The core of the monitoring system is an Arduino microcontroller, selected for its low cost and versatility. A custom program was developed to replicate other gas quantification models, such as the experiment conducted by Shamurad et al. [12]. This approach facilitated efficient data collection and ensured scalability across various experimental conditions. A data-logging module was integrated with the Arduino, featuring an SD-card interface and an internal clock for time-synchronized data recording. The system was configured to store new data every 4 s, providing high-resolution monitoring of pressure dynamics.

The experimental setup included four MPX5700DP pressure sensors arranged on a prototyping board. Each sensor has a measurement range of 0–700 kPa, an output voltage range of 0.2–4.7 V, and an accuracy of ± 1.5% of full scale, according to the manufacturer’s specifications. The sensors feature a typical sensitivity of 6.4 mV/kPa and operate at a 5 V supply voltage. These characteristics make the MPX5700DP suitable for low-pressure gas measurements, ensuring high linearity and stability, which are essential for reliable quantification of gas production in bioreactor experiments. Three of the sensors were connected to identical bioreactors, enabling triplicate measurements to ensure experimental consistency. The fourth sensor was left open to ambient air and served as a reference for atmospheric pressure. All sensors were connected to an Arduino Uno microcontroller via jumper wires. A data logging shield was attached to the Arduino, allowing an SD card to be inserted for storing sensor readings. Additionally, an LCD screen was connected to the system to display pressure readings in real time. A push button on the protoboard was configured to manually trigger data logging. Once activated, the system recorded pressure values from all four sensors every 4 s and saved the data to the memory card, enabling the subsequent construction of gas production curves based on pressure differentials. A schematic diagram of the measurement module, along with a photograph of the fully assembled system, is shown in Fig. 1. Sensor data could also be saved manually via a button integrated into the contact matrix.

**Fig. 2:**
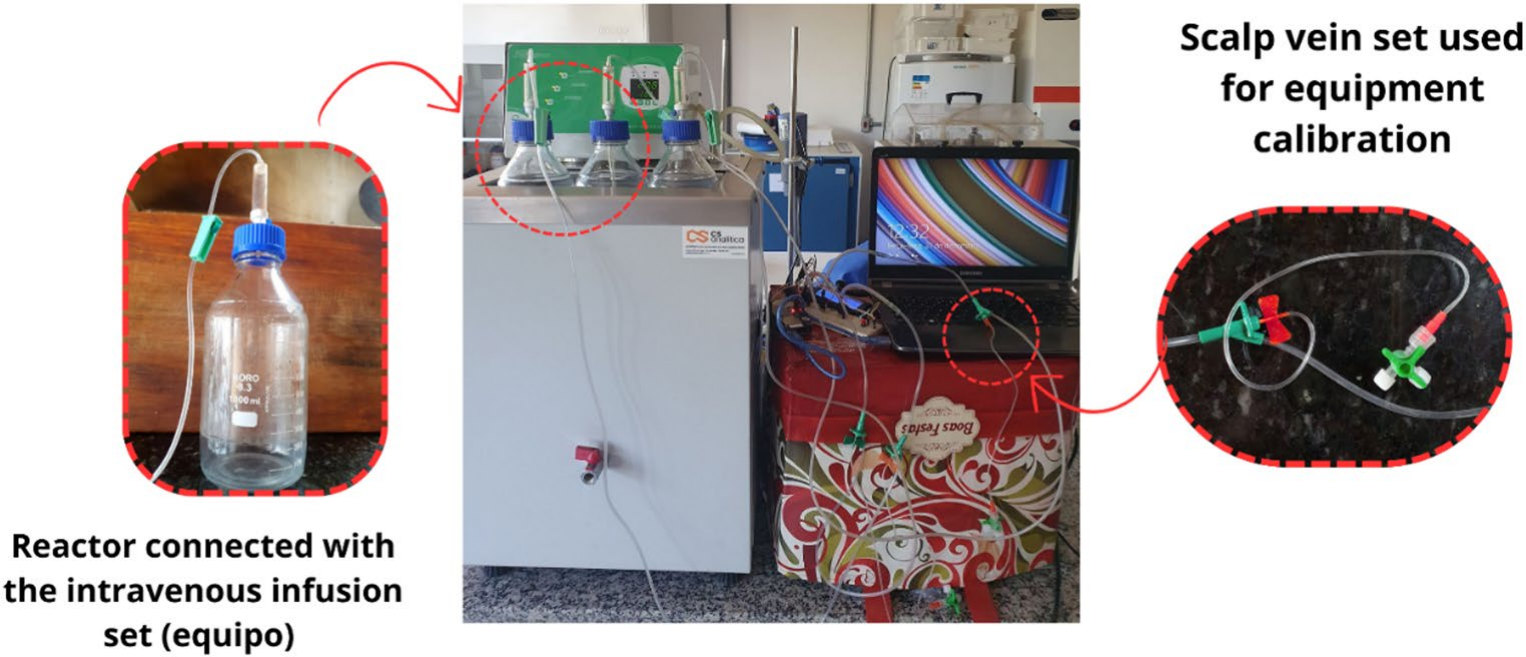
Overview of the experimental test bench used for real-time gas production monitoring

For pressure acquisition inside the bioreactors, each borosilicate flask was fitted with a modified screw cap. A central hole was drilled into each lid, through which an intravenous infusion tube was inserted and permanently sealed with Araldite^®^ adhesive to ensure airtightness. The external end of the hose was connected directly to the readout port of the corresponding pressure sensor. This modification enabled the accurate detection of internal pressure increases due to gas accumulation during biological activity. The integration of the infusion hose ensured stable and secure pressure transmission while minimizing gas leakage. The mechanical configuration of the adapted bioreactors, along with the sensor connections, is illustrated in Fig. 2.

Table 1 summarizes the costs associated with conducting the experiment, highlighting the affordability and feasibility of the developed system. This scalable and cost-effective design provides a reliable solution for real-time monitoring of gas production in laboratory scale bioreactor experiments. In contrast to commercial system, such as the BMP B2 Biogas system, which rarely priced between $6,000 to $10,000 and typically includes basic gas collection flasks and manual or semi-automated volumetric counters, the proposed system integrates pressure sensors (MPX5700), automated data logging, and low-cost, readily available components. The system was designed to ensure accurate and reproducible gas measurement while maintaining low implementation costs. Its modular and open-source architecture enhances scalability and adaptability, allowing straightforward replication. These characteristics make the proposed setup a feasible, reliable, and repeatable solution for laboratory-scale applications.

**Table 1:** Cost comparison between the proposed low-cost gas monitoring system and commercial biogas measurement kits

Item	Average Price in July 2025 (USD)
Arduino System + LCD Display + Logger	$20
4 Sensors MPX5700	$120
3 × 1 L Borosilicate Flasks	$40
Other Items (hoses, glue, etc.)	$20
Total	$200
Commercial Gas Measurement System (Kit BMP B2Biogas) *	$6,000 - $10,000

*Estimated price range based on market data in Brazil (July 2025).

### System calibration and preliminary bench testing

To determine the suitability of the pressure sensors for bench-scale experiments, a comprehensive full-system calibration was performed prior to their use. Each reactor was connected to an intravenous infusion set (equipo), with a scalp vein set (scalp) attached to the lateral outlet of the hose (Fig. 2). With the Arduino code already running, a 50 mL plastic syringe was connected to the scalp adapter, and air was incrementally injected into the system in 10 mL steps, up to a total volume of 300 mL. After each injection, the corresponding pressure variation was recorded directly from the LCD display.

This procedure was conducted individually for all three pressure sensors, each connected to a separate reactor. From the collected data, a linear regression was performed for each sensor to obtain a calibration curve (i.e., a straight-line equation). The output values corresponding to known air volumes were then incorporated into the Arduino code, enabling the system to adjust for sensor-specific calibration offsets. Each sensor underwent three rounds of calibration to ensure consistency and reliability. To statistically evaluate the consistency among the three sensors, the Friedman test was employed. At the end of the calibration process, the minimum detectable gas volume variation (in mL), corresponding to the smallest measurable pressure change, was determined for each sensor, confirming the overall sensitivity of the system.

After calibration, the sensors were subjected to bench testing to verify their performance under realistic conditions and to validate the proposed measurement technique. For these tests, each reactor was filled with 200 mL of water, 1 g of glucose (PA, Synth), and 1 g of biological yeast. This setup used gas biologically generated during the anaerobic fermentation of glucose, simulating conditions typically observed in anaerobic digestion processes and allowing for a realistic evaluation of sensor responsiveness, stability, and consistency under controlled laboratory conditions. The validation approach was designed to evaluate the accuracy, repeatability, and reliability of the developed system by comparing sensor readings across replicates and monitoring gas accumulation over time. The final gas generation data were compared with the stoichiometric reaction of glucose fermentation, as shown in Eq. 1:

1 $$\:{\mathrm{C}}_{\mathrm{6}}{\mathrm{H}}_{\mathrm{12}}{\mathrm{O}}_{\mathrm{6}}\rightarrow{\mathrm{2C}}_{\mathrm{2}}{\mathrm{H}}_{\mathrm{5}}\mathrm{OH+}{\mathrm{2CO}}_{\mathrm{2}}$$

The theoretical generation of carbon dioxide was estimated based on stoichiometry. As the fermentation of 1 mol of glucose produces 2 moles of carbon dioxide, it can be inferred that 0.0055 mol of glucose (equivalent to 1 g of glucose) can generate 0.0111 mol of carbon dioxide. Given that the volume occupied by 1 mol of any gas at *S*TP is 22.4 L, the theoretical estimate of carbon dioxide generation is approximately 249 mL.

To simulate fermentation conditions, the reactors were immersed in a water bath set at 34 °C for 9 h. Pressure measurements were then taken, and the values were normalized to atmospheric pressure. Every 10 min, the pressure data were averaged, and a data point was plotted on the gas generation curve to track the progress of fermentation over time.

Subsequently, the data were fitted to the Gompertz model, which is widely used in studies involving microorganism growth. First introduced by Benjamin Gompertz in 1825, the model has since evolved and has been parameterized by various researchers, expanding its applicability. One such parameterization was proposed by Zwietering et al. [13]. The Gompertz model has also been successfully applied in BMP assays, as demonstrated by Budiyono et al. [14] and Shamurad et al. [12].

The parameterization of the model is given by Eq. 2, which corresponds to the modified Gompertz model, widely applied in biogas production kinetics, as shown by Lay et al. [15] and Guo & Wang [10]:

2 $$\:\mathrm{Y=\:P*}\mathrm{exp}\left\{\mathrm{-exp}\left[\frac{\mathrm{Rm*e(1)}}{\mathrm{P}}\text{}\left(\text{}\lambda\mathrm{-t}\right)\mathrm{+1}\right]\right\}$$

where $\:Y$ represents the cumulative gas production at time $\:t$, $\:P$ is the maximum methane potential (mL), Rm is the gas production rate per hour (mL/h), *e(1)* is a mathematical constant (2.718) and λ is given by the lag phase, referring to the onset of gas generation in the system, expressed in hours (h).

Measurement variability was assessed by calculating the standard deviations among triplicates and across the three sensors, providing an estimate of measurement uncertainty. To evaluate sensors consistency, repeated-measures ANOVA was performed on the mean values of the triplicates, allowing a statistical comparison of the three sensors under controlled laboratory conditions.

### Central composite design (CCD) assay development

After data validation, a test was performed using the Central Composite Design (CCD) to evaluate whether the system is sensitive to variations in response variables as a function of the independent factors. The same apparatus developed during the calibration stage was used, with modifications only to the design of the assays. The number of assays, based on the variations of the factor “k”, is determined by the following equation:

3 $$\:\mathrm{Number\:of\:assays=\:r*}{\mathrm{2}}^{\mathrm{k}}\mathrm{+}\mathrm{cp}$$

where “r” refers to the number of genuine repetitions in the “k” factors, and “cp” is given by the number of repetitions at the central points, as described by Rodrigues and Iemma [16]. In this study, the value of “r” was 1, “k” was equal to 2, and “cp” was equal to 3, totaling 7 assays.

The reactor feed composition consisted of 200 mL of water, 1 g of glucose and 1 g of commercial biological yeast. Each assay was conducted over a duration of 12 h. The temperature of the water bath and the pH of the solutions were adjusted according to the conditions specified in Table 2.

**Table 2:** Setup for the statistical design performed

Assay number	pH	Temperature (°C)
1	5.5	20
2	5.5	40
3	8.5	20
4	8.5	40
5	7	30
6	7	30
7	7	30

After data collection, the Gompertz model was applied to the dataset. Subsequently, the data were analyzed using an analysis of variance (ANOVA). ANOVA is one of the most widely used methods for analyzing experimental results. This technique decomposes the total variability observed in a response variable, based on the chosen statistical model, and evaluates the “statistical significance of the variation” factors [17].

## Results and discussion

### Sensor calibration

**Fig. 3:**
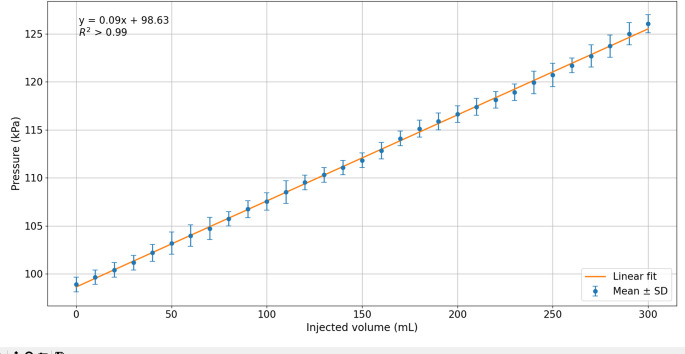
Relationship between injected volume and measured pressure obtained during sensor calibration using an Arduino-based acquisition system. Data are presented as mean values with standard deviation error bars (*n* = 3). The solid line represents the linear regression, indicating high linearity (R² > 0.99)

**Fig. 4:**
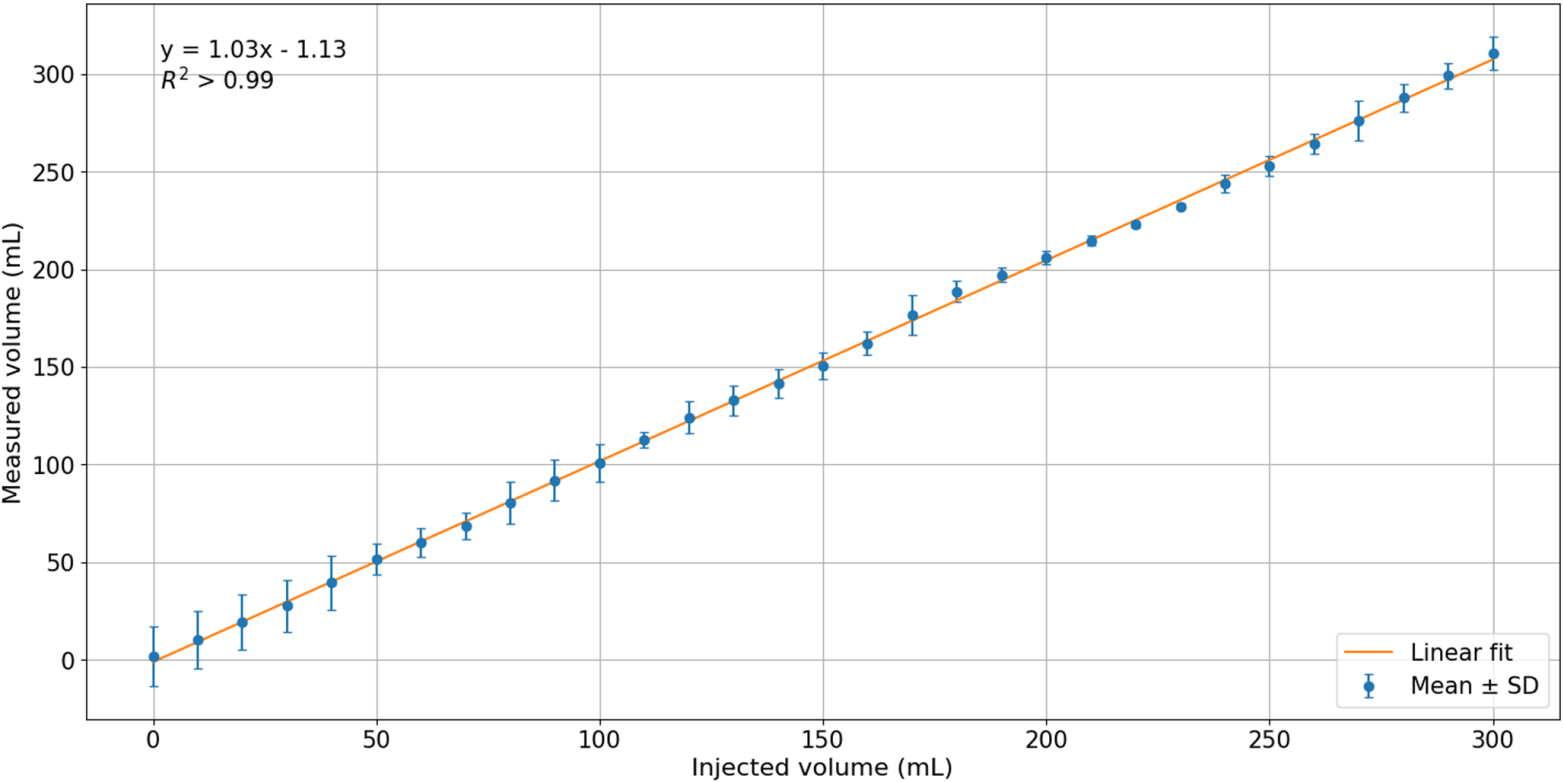
Relationship between injected volume and measured volume during sensor calibration using an Arduino-based acquisition system. Data are presented as mean values with standard deviation error bars (*n* = 3). The solid line represents the linear fit (R² > 0.99)

Calibrating pressure sensors by comparing input volume with measured volume is essential to ensure accurate, reliable, and safe measurements, and must be conducted before testing under controlled conditions. This process validates sensor performance, enhances data quality, and minimizes errors that could compromise experimental results or system safety. Calibration also supports accurate modeling by aligning sensor readings with theoretical or empirical expectations [18]. The comparison between the known input volume and the measurements from sensors 1, 2, and 3 revealed no significant discrepancies, highlighting the reliability and precision the sensors. As shown in Figs. 3 and 4, the calibration curves for pressure sensors 1, 2, and 3 demonstrated strong linearity, with R² values greater than 0.99, confirming the excellent fit of the linear adjustments. Furthermore, the slopes of these calibration curves were very close to 1, indicating that the measured volumes closely matched the input volumes and reinforcing the accuracy and consistency of the sensors across the tested range. Factors such as system resolution, with a minimum detectable volume change of approximately 1 mL, also contributed to the high accuracy observed during calibration.

The mean values of the sensors were calculated, and the standard deviations were used to quantify the variability among sensors (Figs. 3 and 4). These standard deviations are displayed as error bars in the calibration plot, providing a clear visualization of the system reproducibility. Given that the measurements did not follow a normal distribution, the Friedman test was employed to compare the three sensors. No statistically significant differences were observed (*p* > 0.05), confirming consistent performance and effective calibration. The combined results from the statistical analysis and the small deviations among sensor readings demonstrate high repeatability and reproducibility of the measurement system. Furthermore, the measurement uncertainty was explicitly quantified using the standard deviation among repeated sensor readings, providing a reliable estimate of the confidence interval for the measured gas volumes and supporting the traceability of the method.

### Preliminary bench testing

**Fig. 5:**
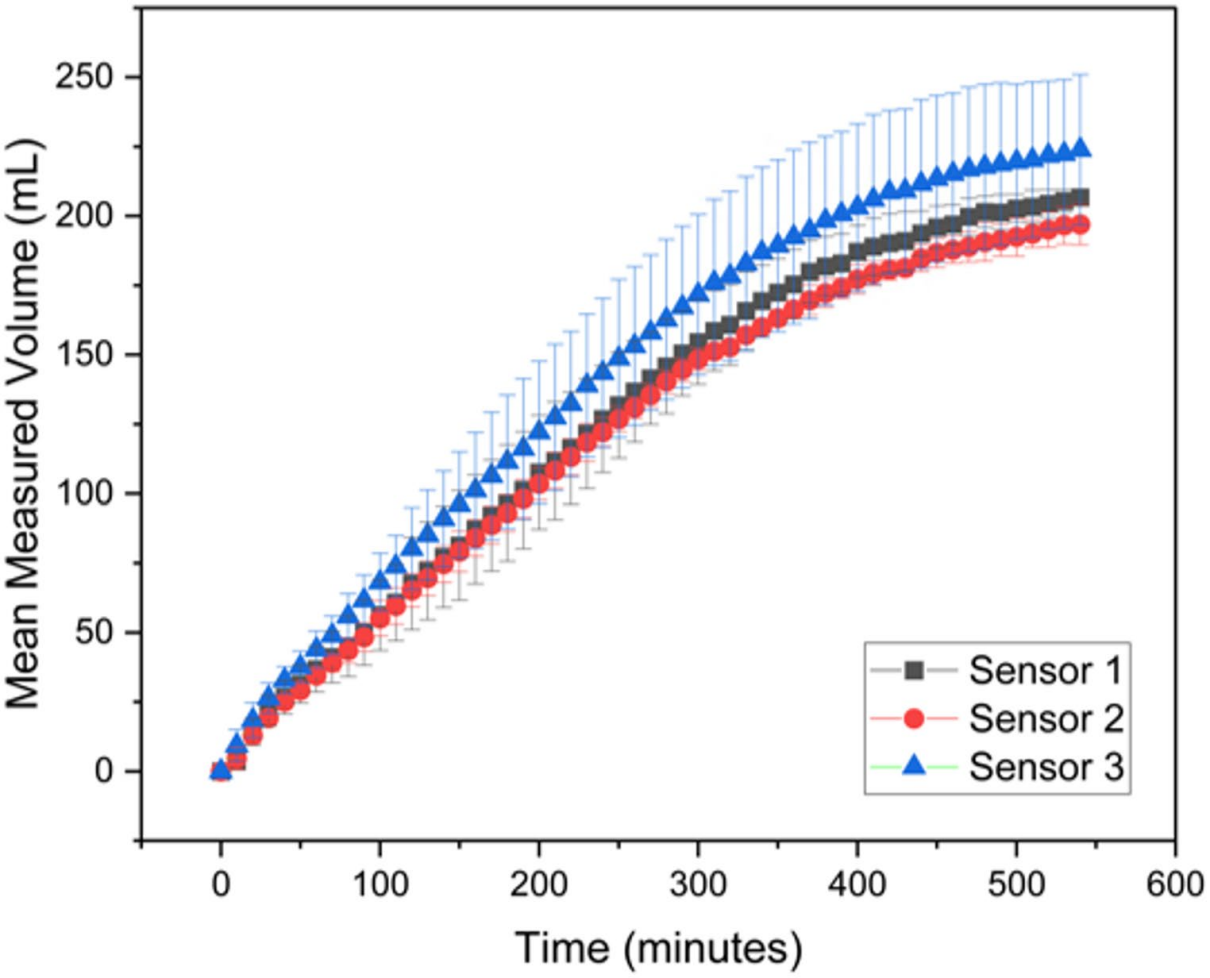
Mean gas volume measurements for eac3h of the three pressure sensors (1, 2, and 3) obtained from triplicate measurements using the Arduino-based acquisition system. Error bars represent one standard deviation among the triplicates for each sensor

After calibration, a preliminary bench test was conducted to evaluate the performance of the three pressure sensors under controlled laboratory conditions. Each reactor was filled with 200 mL of water, 1 g of glucose PA, and 1 g of biological yeast, and the experiment was run for 540 min. Each sensor was tested in triplicate, and mean values were calculated. Standard deviations were used to quantify variability among the triplicates and sensors, providing a clear assessment of measurement uncertainty. These standard deviations are displayed as error bars in the calibration plot (Fig. 5), illustrating the system reproducibility. Since the measurement data followed a normal distribution, a repeated-measures ANOVA was performed on the mean values of the triplicates to compare the three sensors. No statistically significant differences were observed (*p* > 0.05), confirming consistent performance, effective calibration, and high repeatability and reproducibility. Overall, the combined results from the statistical analysis, low variability among sensor readings, and visualization of standard deviations demonstrate the reliability and traceability of the measurement system.

Preliminary bench testing revealed final gas production values of 201 ± 8.2 mL, 197 ± 8.3 mL, and 224 ± 9.2 mL for sensors 1, 2, and 3, respectively. The gas production measurements from sensors 1 and 2 exhibited significant similarity, indicating consistent fermentation conditions in these reactors. However, the gas production recorded by sensor 3 was approximately 15% higher than that of sensors 1 and 2 (Fig. 5). Despite this discrepancy, a statistical analysis performed on the triplicate measurements for each sensor showed no statistically significant differences, indicating that the observed variation was within the expected experimental uncertainty. This discrepancy is likely due to experimental errors within the system, which could be related to inaccuracies in the weighing of glucose and/or yeast, or to the position of reactor 3 in the water bath. Unlike reactors 1 and 2, reactor 3 was positioned a few centimeters closer to the heating element in the water bath. Given that the water bath lacked an agitation system to ensure temperature homogeneity, it is probable that reactor 3 experienced slightly higher temperatures, which in turn accelerated fermentation. Temperature plays a crucial role in biogas production, as the anaerobic degradation of organic substrates is temperature dependent. This makes temperature one of the most important operational parameters to control during fermentation processes. The observed variation in gas production emphasizes the need for homogeneous temperature distribution in experimental setups to ensure accurate and reproducible results [19, 20].

Despite the observed discrepancies, all experimental values remained below the theoretical total glucose fermentation value of 249 mL for 1 g of substrate, achieving approximately 80% of the theoretical potential. This result is consistent with the findings of Angelidaki et al. (2009) [21], who demonstrated that in BMP (Biochemical Methane Potential) assays, part of the substrate is utilized for bacterial biomass synthesis, thereby reducing gas yields compared to the theoretical maximum. These findings indicate that the experimental setup performed as expected and validate the reliability of the measurements. While longer observation periods can provide a more complete picture of fermentation kinetics, the 12-hour duration used in this study is sufficient to capture the primary gas production dynamics for glucose fermentation in benchtop reactors. This period covers the main phase of biogas generation, which is critical for validating the performance of the pressure sensors and the measurement system. Extending the observation time would mainly capture slower, residual gas production, which is not essential for the current validation and calibration objectives. To further investigate the fermentation dynamics, the experimental data were fitted to the Gompertz model. The resulting parameters, including the lag phase duration (λ), maximum gas generation rate (R_m_), and final gas production (P), are summarized in Table 3. These kinetic parameters provide valuable insights into the performance of the reactors and help to elucidate the observed differences in gas production, reinforcing the importance of controlling experimental conditions to minimize variability.

**Table 3:** Average sensor readings applied to the Gompertz model

Sensor average	Lag (Y_1_, h)	*R*_m_ (Y_2_, mL/h)	*P* (Y_3_, mL)
1	0	32.1	213.9
2	0.1	32.5	209.4
3	0	37.0	237.4

As shown in Table 3, no lag phase was observed in the assays. This outcome may be explained by the nature of the materials used in anaerobic reactions. The experiment employed analytical-grade glucose P.A. and commercial biological yeast, which likely led to near-instantaneous gas generation. Under these conditions, microorganisms may not require significant acclimation time, potentially explaining the absence of a detectable lag phase. This characteristic may have influenced the fitting of the Gompertz model with respect to the lag phase parameter. Regarding the maximum gas generation rate (Rm), the values across the three sensors were very similar, indicating consistent degradation rates in all reactors. The estimated final gas production (P), based on the Gompertz model, was close to the theoretical maximum value of 249 mL but did not exceed it at any point. This is a favorable result, as it aligns with the expected behavior of the system. Moreover, the P values closely matched the average gas production values obtained experimentally, further confirming the accuracy of the system calibration. To validate the developed system and support the reliability of the data, a statistical analysis using a Central Composite Design (CCD) was conducted. This approach provided additional confirmation of the observed trends and demonstrated the robustness of the experimental setup.

### Sensitivity assays (CCD)

**Fig. 6:**
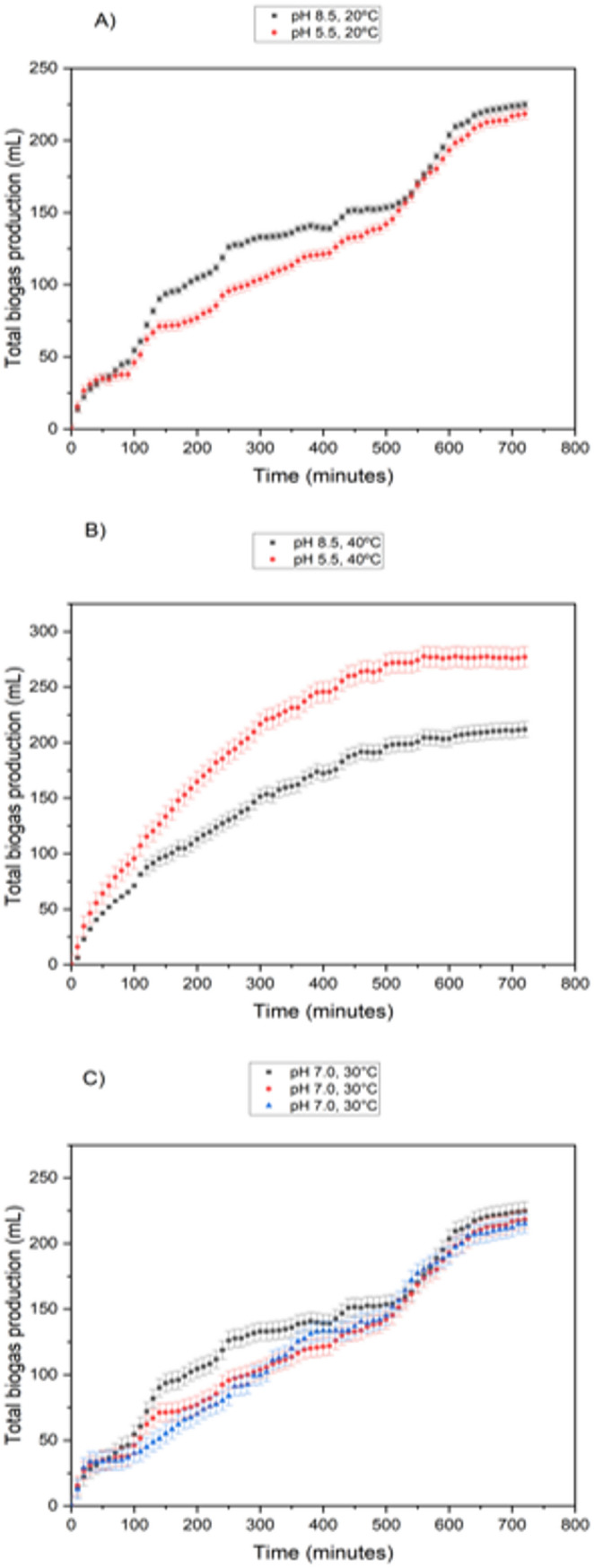
Total biogas production in assays 1 and 3 (**A**), 2 and 4 (**B**), 5,6 and 7 (**C**)

A specific simulated scenario was developed, incorporating data collection over 12 h, based on the Central Composite Design (CCD). This approach enabled the systematic evaluation of variables and their interactions, providing a robust framework for further statistical analysis using analysis of variance (ANOVA). The experimental results were further modeled and adjusted using the Gompertz equation, which is widely recognized for its ability to describe microbial growth and biogas production kinetics [10]. Figure 6 illustrates the assay outcomes, highlighting the relationship between the tested variables and the observed responses. Notably, the results emphasize the impact of temperature and pH on biogas production, revealing significant variations. Low temperatures negatively affected the performance of both the acidogenic and methanogenic phases, leading to reduced biogas generation. In contrast, higher biogas production efficiency was observed at moderate temperatures above 25 °C [19, 22]. Additionally, pH variations influenced both methane production and methane content in the biogas [19], further demonstrating its critical role in optimizing the process. These findings validate the effectiveness of the experimental design in identifying key factors influencing biogas production and confirm the suitability of the Gompertz model for describing the observed dynamics.

The final gas generation for assay 1 was 94.8 ± 3.5 mL, while for assay 3, it was 65.1 ± 2.6 mL. Up to the point of interruption, gas production was higher in the acidic pH solution compared to the alkaline solution. However, this does not confirm that acidic pH results in greater overall gas production, as the gas volume generated corresponded to less than 40% of the theoretical total for the fermentation of 1 g of glucose. This indicates that gas generation likely would have continued if the fermentation had reached completion. Additionally, at 20 °C, slower gas production limited the interpretability of the 12-hour observation period.

In contrast, assay 2 generated 277 ± 9.2 mL of gas, while assay 4 produced 212 ± 6.9 mL. The gas volume in assay 2 slightly exceeded the theoretical value for 1 g of glucose (249 mL), likely due to experimental errors, such as inaccuracies in preparation, sensor coupling issues, or partial degradation of the yeast cells.

For assays 5, 6, and 7, the final gas volumes were 225 ± 7.0 mL, 218 ± 6.9 mL, and 215 ± 7.2 mL, respectively. Despite an initial variation in assay 5, the results aligned with the others, falling within the expected error range. These values were consistent with theoretical predictions, indicating that most glucose was fermented, although some residual substrate likely remained for further conversion.

To perform the ANOVA, it was necessary to define the system’s independent and dependent variables. The independent variables were pH (x₁) and temperature (x₂, °C), while the dependent variables were the lag phase duration (Y₁, h), the maximum gas generation rate (Y₂, mL/h), and the final gas production potential (Y₃, mL) obtained from fitting the Gompertz model. The data used for the ANOVA are presented in Table 4.

**Table 4:** Variables and results used in ANOVA analysis

Assay	pH (x_1_)	Temp (x_2_, °C)	Lag (Y_1_, h)	*R*_m_ (Y_2_, mL/h)	*P* (Y_3_, mL)
1	5.5	20	0.6	11.3	101.1
2	5.5	40	0	44.3	283.7
3	8.5	20	1.6	8.2	70.3
4	8.5	40	0	28.1	217.9
5	7	30	0	19.0	324.3
6	7	30	0	18.4	462.6
7	7	30	0	18.4	268.5

The results of the ANOVA analysis indicate that the lag phase duration does not have a statistically significant relationship with the Gompertz model under the experimental conditions used. This conclusion is supported by an R² value of 54.0% and a p-value above the acceptable threshold (*p* > 0.05). A similar observation was made during the calibration phase when the lag phase was calculated. Likewise, the final gas production potential (P) showed an R² of 25.3% and a p-value greater than 0.05 (*p* > 0.05), further suggesting that the final gas generation in the simulated scenario was not significantly influenced by the conditions outlined in the experimental design.

This lack of significance may be attributed to the slow kinetics of the fermentation process at moderate temperatures, as these conditions require longer durations for measurable changes to occur. The 12-hour experimental period may have been insufficient to capture a complete dataset, leading to premature stabilization of the curve and potentially affecting the reliability of the Gompertz model’s parameters. Consequently, the early termination of the experiment likely limited the statistical power of the ANOVA, resulting in non-significant findings.

To address this limitation, it is recommended that future experiments extend the duration of the assays. A longer observation period would likely provide a more complete representation of the fermentation process, allowing for more robust parameter estimation and improved statistical significance. Such adjustments would enable a better understanding of the relationship between the model parameters and the experimental conditions.

Despite these limitations, it is important to emphasize that the primary focus of this study was the creation and validation of a low-cost measurement system. While the short experimental duration may have influenced the statistical outcomes, it does not diminish the success of the proposed system. The methodology developed in this study remains a promising tool for monitoring fermentation processes and can be further refined in future studies with extended assay durations. The maximum gas generation rate (R_m_) demonstrated satisfactory p-values (*p* < 0.05), with the system achieving an R² value of 96.2%. This indicates that Rm is significantly influenced by variations in pH (x₁) and temperature (x₂). Consequently, it is possible to formulate an equation to maximize Rm as a function of x₁ and x₂. For this purpose, Eq. 4 is applied:

4 $$\:{Y}_{2}=\:\mathrm{21,08}-\mathrm{4,82}\:{x}_{1}+\:\mathrm{13,24}\:{x}_{2}-\mathrm{3,28}\:{x}_{1}{x}_{2}$$

Here, R_m_ corresponds to Y₂, with x₁ representing any pH value within the range of 5.5 to 8.5, and x₂ denoting any temperature value between 20 and 40 °C.

**Fig. 7:**
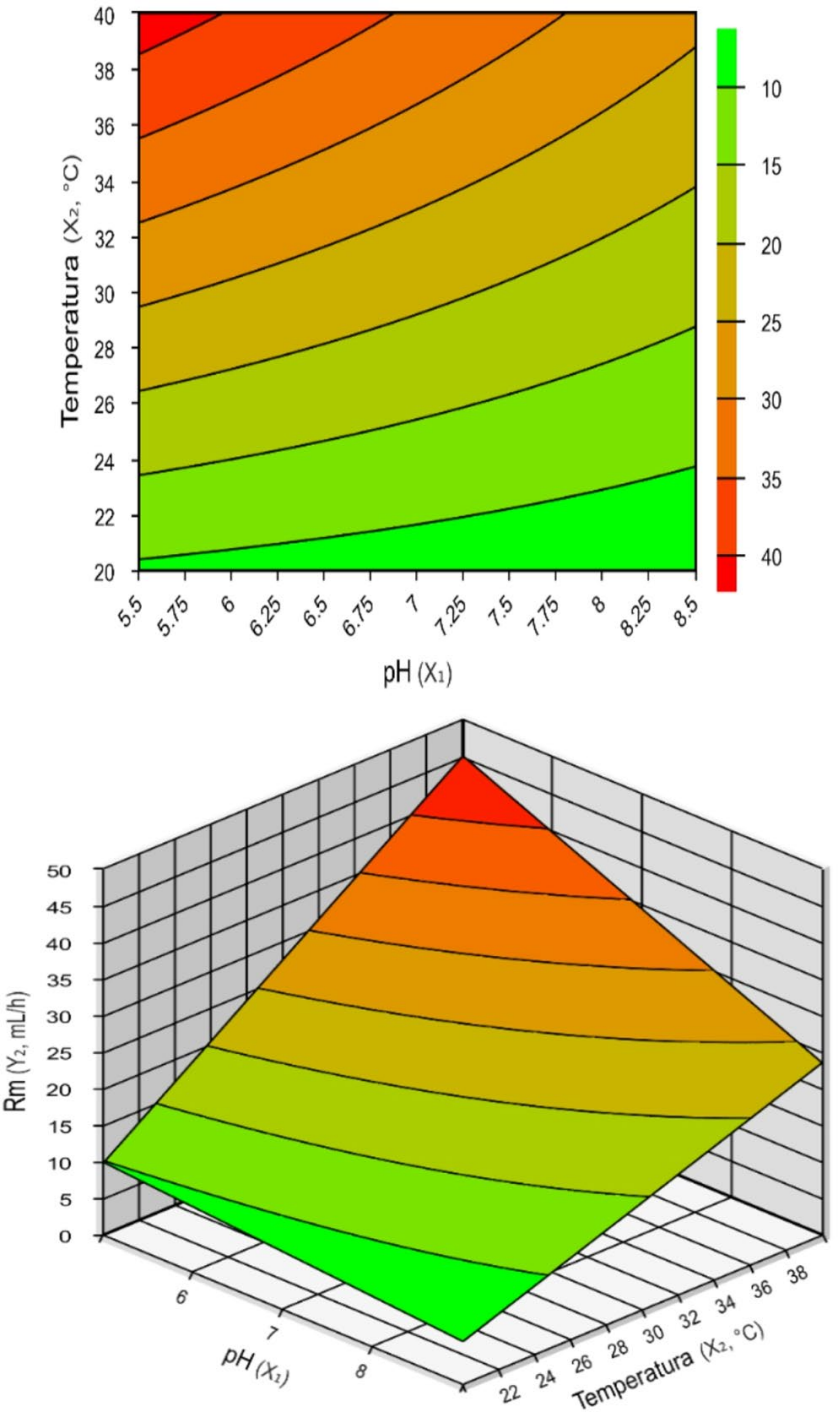
Response surface showing the relationship between pH, temperature (°C), and R_m_

Based on the results, a response surface plot was constructed to illustrate the relationship between the independent variables, x₁ (pH) and x₂ (temperature), and their effect on R_m_. This surface, shown in Fig. 7, highlights the connection between these factors and their influence on the maximum gas generation rate. The surface aligns well with the predictions made by Eq. 4, reinforcing the validity of the model and its ability to describe the system behavior across the tested conditions.

The improvements to the sensor system enhance its usability, reliability, and accuracy, directly supporting the construction of the response surface. By providing real-time, precise measurements of pH and temperature, the sensor ensures that the data accurately reflects the experimental conditions. This consistency strengthens the confidence in the surface’s ability to predict system behavior and confirms that the observed trends align with the theoretical model. Ultimately, the improved sensor setup enables more accurate modeling and better decision-making for optimizing fermentation processes.

### Sensor performance and applicability

The developed gas measurement device demonstrated satisfactory performance under realistic laboratory conditions, as evidenced by the Central Composite Design (CCD) results. The sensors consistently captured reliable data throughout the study, responding effectively to variations in temperature and pH, which highlights both their sensitivity and stability. The system also exhibited strong linearity in data acquisition, reinforcing the robustness of the measurements. By automating the measurement process, the device reduces labor requirements and potential human error, enabling continuous monitoring and reliable data collection under varying operational conditions.

Compared to traditional gas measurement methods, the developed device presents several clear advancements. It is fully automated, cost-effective, and highly sensitive, offering a practical solution for laboratories with limited resources. Successful calibration and validation across a range of pH values and temperatures confirm the system’s accuracy, reproducibility, and robustness. By explicitly quantifying measurement uncertainty, error propagation, and system performance, this work provides a methodologically rigorous and reproducible approach, representing a significant contribution to measurement science.

To contextualize these results, several studies in literature have explored similar approaches. The system developed by Araujo et al. [5], provides reliable volumetric gas flow measurements using Arduino-based components and open-source software; however, they are limited to total gas flow and lack automation or responsiveness to environmental factors. Volumetric gas meters by Pereda et al. [23] and Liu et al. [24] offer reliable laboratory-scale monitoring, with Pereda’s system using optical detection to reduce errors from vibrations and minimize CO₂ losses, while Liu’s device operates on liquid displacement with improved counting logic and real-time data display. However, both systems involve greater hardware complexity and require specialized technical expertise.

Recent research has increasingly focused on developing low-cost sensors for gas detection. In this context, Fakra et al. [25] proposed a method using an adaptable capsule with MQ-4 and MQ-8 sensors to measure high concentrations of methane and hydrogen. Their approach involved diluting the gas in a known volume of air and testing three setups: an airtight chamber, direct injection in an open environment, and a partially closed capsule. Comparisons showed that the airtight chamber provided the best repeatability, though with limited linearity, while direct injection offered better linearity but poor repeatability. The partially closed capsule achieved the best overall performance, with high linearity (R² = 0.997 for CH4 and R² = 0.947 for H2) and acceptable measurement ranges (up to 20% CH4 and 13.3% H2). This simple and low-cost technique enables the characterization of combustible gases in remote areas, allowing local operators of biomass valorization systems to monitor and optimize their installations without relying on expensive conventional devices.

Complementing this approach, Nagahage et al. [26] developed a continuous methane monitoring system using multiple low-cost sensors (TGS 2611 and MQ-4) with an automated gas sampling unit and a cloud-based data acquisition platform. The study verified consistency, repeatability, and reproducibility by measuring both high- and low-concentration methane samples, with normalized root-mean-square errors (NRMSEs) confirmed against a gas chromatograph. Laboratory-scale anaerobic digesters were continuously monitored for two months, demonstrating the potential for reliable field-level methane detection. This work illustrates how integrating multiple low-cost sensors with automation can overcome the limitations of individual sensors, enabling continuous, accurate, and practical monitoring applications.

The study by Vidal et al. [27] introduces an IoT-based biogas pressure measurement device developed using an Arduino microcontroller to enhance the accuracy and reliability of batch anaerobic digestion tests, such as Biochemical Methane Potential (BMP) and Specific Methanogenic Activity (SMA) assays. The research demonstrates that the conventional Manual Manometric (MM) method tends to underestimate biogas production due to gas losses during pressure measurements, with an average loss of 50.7 ± 12.9 mbar per measurement. In contrast, their IoT-based system enables continuous, real-time monitoring, early detection failure, efficient data management, and more accurate observation of biogas production dynamics, including acidogenic and methanogenic phases. Compared to the IoT-based device developed by Vital et al. (2025), our system not only monitors biogas pressure in real time but also integrates pH and temperature sensing, offering greater environmental responsiveness, enhanced measurement accuracy, and improved reproducibility, making it a more versatile and reliable tool for laboratory-scale anaerobic digestion studies.

In comparison, the system developed in this study integrates the strengths of both approaches while overcoming their limitations. Although the current work focused on laboratory-scale applications, the system’s modular design and robust performance indicates its potential for future adaptation to pilot- or industrial-scale bioprocess monitoring. By emphasizing the device’s measurement performance rather than specific applications, this work advances the broader field of measurement science, offering a scalable, accessible, and reliable solution for accurate gas quantification.

## Conclusion

Based on the tests performed, the developed method for gas quantification proved to be effective, reliable, and robust. The system performed satisfactorily during calibration and validation, successfully achieving the primary objective of this study: developing and evaluating a device for gas measurement under controlled laboratory conditions. Statistical analysis showed that in 90% of cases, the results aligned with predictions from the Central Composite Design (CCD) across operational conditions with pH values between 5.5 and 8.5 and temperatures from 20 to 40 °C. These results demonstrate the consistency, sensitivity, and stability of the device under varying conditions. Compared to conventional methods, the system provides high automation, enhanced precision, and ease of use, making it a practical and cost-effective solution for laboratory-scale gas measurement. Moreover, the study includes an explicit uncertainty analysis, addressing error propagation, repeatability, and reproducibility, thereby reinforcing the metrological reliability of the system and highlighting its contribution to measurement science.

## Data Availability

No datasets were generated or analysed during the current study.
